# Neurodevelopment correlates with gut microbiota in a cross-sectional analysis of children at 3 years of age in rural China

**DOI:** 10.1038/s41598-021-86761-7

**Published:** 2021-04-01

**Authors:** Sarah E. Rothenberg, Qiurong Chen, Jian Shen, Yanfen Nong, Hua Nong, Eva P. Trinh, Fred J. Biasini, Jihong Liu, Xiaoyun Zeng, Yunfeng Zou, Fengxiu Ouyang, Susan A. Korrick

**Affiliations:** 1 College of Public Health and Human Sciences, Oregon State University, 103 Milam Hall, Corvallis, OR 97331 USA; 2 Key Laboratory of Systems Biomedicine (Ministry of Education), Shanghai Center for Systems Biomedicine, Shanghai Jiao Tong University, Shanghai, China; 3 Maternal and Child Health Hospital, Daxin County, China; 4 Department of Psychology, University of Alabama at Birmingham, Birmingham, AL USA; 5 Department of Epidemiology and Biostatistics, Arnold School of Public Health, University of South Carolina, Columbia, SC USA; 6 Department of Epidemiology and Statistics, School of Public Health, Guangxi Medical University, Nanning, Guangxi China; 7 Department of Toxicology, School of Public Health, Guangxi Medical University, Nanning, Guangxi China; 8 Ministry of Education and Shanghai Key Laboratory of Children’s Environmental Health, Xinhua Hospital, Shanghai Jiao Tong University School of Medicine, Shanghai, China; 9 Channing Division of Network Medicine, Department of Medicine, Brigham and Women’s Hospital and Harvard Medical School, Boston, MA USA; 10 Department of Environmental Health, Harvard T.H. Chan School of Public Health, Boston, MA USA; 11 Present Address: Centers for Disease Control and Prevention, Atlanta, GA USA

**Keywords:** Neuroscience, Cognitive neuroscience, Microbial communities, Microbiome

## Abstract

We investigated cross-sectional associations between children’s neurodevelopment and their gut microbiota composition. Study children (36 months of age) lived in rural China (n = 46). Neurodevelopment was assessed using the Bayley Scales of Infant Development, 2nd Edition, yielding the Mental Developmental Index (MDI) and Psychomotor Developmental Index (PDI). Children's gut microbiota was assessed using 16S rRNA gene profiling. Microbial diversity was characterized using alpha diversity patterns. Additionally, 3 coabundance factors were determined for the 25 most abundant taxa. Multivariable linear regression models were constructed to examine the relationships between Bayley scores (MDI and PDI) and children's gut microbiota. In adjusted models, MDI and PDI scores were not associated with alpha diversity indices. However, in adjusted models, MDI and PDI scores were positively associated with the first coabundance factor, which captured positive loadings for the genera *Faecalibacterium*, *Sutterella,* and *Clostridium* cluster XIVa. For an interquartile range increase in the first coabundance factor, MDI scores increased by 3.9 points [95% confidence interval (CI): 0, 7.7], while PDI scores increased by 8.6 points (95% CI 3.1, 14). Our results highlight the potential for gut microbial compositional characteristics to be important correlates of children's Bayley Scales performance at 36 months of age.

## Introduction

Increasing evidence supports a central role for the gut microbiome in neurodevelopment, with communication along the gut-brain axis postulated as one plausible mechanism^1,2^. This association may be especially important during the first 3 years of life, when children's brains are rapidly developing^3^, and when their gut microbiomes are changing, as their diets become established^4,5^.

Only a few studies have examined the role of the gut microbiota composition in children's neurodevelopment among typically developing children. Among 309 children participating in a multi-site study (Massachusetts, Missouri, and California, USA), associations were observed between children's gut microbiota (analyzed at 6 months of age) and communication, personal and social, and fine motor skills, which were assessed using the Ages and Stages Questionnaire, 3rd edition, at age 3 years^6^. Among 89 children in North Carolina, USA, gut microbial diversity (assessed at age 1 year) was associated with children's cognitive performance, including overall cognition, visual reception, and expressive language, assessed using the Mullen Scales of Early Learning, at age 2 years^7^. Among 77 children approximately 2 years of age in Ohio, USA, temperament was assessed by maternal report using the Early Childhood Behavior Questionnaire^8^. Temperament, a correlate of children's cognitive development, was associated with the diversity and composition of the children's gut microbiota (analyzed at the same time point)^8^. Although limited in number, these studies suggest that the gut microbiota may play a role in children's cognitive development.

Since 2013, we have investigated associations between prenatal methylmercury exposure through maternal rice ingestion and children's subsequent neurodevelopment, among participants in a birth cohort study in rural China^9–11^. In this population, prenatal methylmercury exposure is considered low-level, compared to most studies among fish consumers^9–11^. For the current cross-sectional analysis in a subset of the cohort, we investigated whether children's gut microbiota was associated with neurodevelopmental outcomes at 36 months of age, assessed using the Bayley Scales of Infant Development, 2nd edition (BSID-II)^12^.

## Results

### Study participants

Of 398 mother-infant pairs enrolled in our study, 196 participants (49%) returned at 36 months and completed BSID-II assessments, including 46 participants (23%), who collected their child's stool sample (Table 1). Among children who donated a stool sample, household income was higher, fewer reported illnesses in the previous 12 months, a higher percentage of children ingested fish within the previous 24 h, and Mental Developmental Index (MDI) scores were higher, compared to children who did not collect a stool sample (Table 1).

**Table 1:** Comparison of maternal/child characteristics between children who provided a stool sample and those who did not among 196 children with Bayley testing at 36 months. ^a^P-values are for comparisons between participants providing a stool sample, and those that did not. For categorical variables, p-values are for chi-squared test or Fisher's exact test between non-missing categories, and for continuous variables, p-values are for the Wilcoxon rank sum test: *p < 0.05, **p < 0.01, ***p < 0.001. ^b^BMI for Asian populations: underweight (BMI < 18.5 kg/m^2^), normal weight (18.5 kg/m^2^ ≤ BMI < 23 kg/m^2^), overweight (23 kg/m^2^ ≤ BMI < 27.5 kg/m^2^), and obese (BMI ≥ 27.5 kg/m^2^)^54^. ^c^Workers include: civil servant, white-collar worker, skilled worker, unskilled worker, and shopkeeper. ^d^Median breastfeeding duration based on 12-month and 36-month responses (n = 332 mothers). ^e^Illnesses and symptoms queried included upper respiratory illness, lower respiratory illness, difficulty breathing, diarrhea, vomiting, fever, and rash. Children who donated a stool sample did not have any reported rash or difficulty breathing. ^f^Other meat includes sheep, beef, chicken, or duck. *BMI* body mass intake, *MDI* Mental Developmental Index, *PDI* Psychomotor Developmental Index, *RMB* ren min bi = Chinese currency.

	All (n = 196) n (%) or median (range)	Provided stool sample		p-value^a^
		Yes (n = 46, 23%) n (%) or median (range)	No (n = 150, 77%) n (%) or median (range)	
**Questionnaire responses upon enrollment**				
Mother's age (years)				
Age < 20	15 (8)	3 (7)	12 (8)	0.93
20 ≤ Age < 30	109 (56)	27 (59)	82 (55)	
30 ≤ Age < 45	72 (37)	16 (35)	56 (37)	
Mother's prepregnancy BMI^b^				
Underweight	54 (28)	11 (24)	43 (29)	0.27
Normal weight	107 (55)	23 (50)	84 (56)	
Overweight or obese	35 (18)	12 (26)	23 (15)	
Mother's education completed				
< High School	151 (77)	37 (80)	114 (76)	0.95
High School	29 (15)	6 (13)	23 (15)	
University	12 (6)	3 (7)	9 (6)	
Missing	4 (2)	0 (0)	4 (3)	
Mother's occupation				
Farmer	144 (73)	34 (74)	110 (73)	0.56
Worker^c^	20 (10)	6 (13)	14 (9)	
Unemployed	22 (11)	6 (13)	16 (11)	
Other	6 (3)	0 (0)	6 (4)	
Missing	4 (2)	0 (0)	4 (3)	
Household monthly income (RMB)				
Income < 2000	119 (63)	22 (48)	97 (65)	0.04*
2000 ≤ Income < 5000	51 (26)	17 (37)	34 (23)	
Income ≥ 5000	10 (5)	4 (9)	6 (4)	
Missing	16 (8)	3 (7)	13 (9)	
Cesarean birth				
No	151 (77)	34 (74)	117 (78)	0.56
Yes	45 (23)	12 (26)	33 (22)	
Child sex				
Male	108 (55)	28 (61)	80 (53)	0.37
Female	88 (45)	18 (39)	70 (47)	
Gestational age (weeks)				
Gestational age < 37	6 (3)	1 (2)	5 (3)	0.96
37 ≤ Gestational age < 39	70 (36)	17 (37)	53 (35)	
39 ≤ Gestational age < 41	105 (54)	26 (57)	79 (53)	
Gestational age ≥ 41	13 (7)	2 (4)	11 (7)	
Missing	2 (1)	0 (0)	2 (1)	
**Questionnaire responses at 36 months**				
Primary caregiver				
Mother	118 (60)	29 (63)	89 (59)	0.89
Father	26 (13)	6 (13)	20 (13)	
Grandparent	52 (27)	11 (24)	41 (27)	
Breastfeeding duration > median (8.5 months)^d^				
No	94 (47)	20 (43)	74 (50)	0.46
Yes	101 (53)	26 (57)	75 (50)	
Missing	1 (< 1)	0 (0)	1 (< 1)	
Child attends preschool				
No	74 (38)	14 (30)	60 (40)	0.24
Yes	122 (62)	32 (70)	90 (60)	
Reported any child illness in the previous 12 months^e^				
No	30 (15)	13 (28)	17 (11)	0.005**
≥ 1 illness (range: 1–4)	166 (85)	33 (72)	133 (89)	
Reported child fever in the previous 12 months				
No	145 (74)	37 (80)	108 (72)	0.25
Yes	51 (26)	9 (20)	42 (28)	
Reported child upper-respiratory illness in the previous 12 months				
No	55 (28)	19 (41)	36 (24)	0.02*
Yes	141 (72)	27 (59)	114 (76)	
Reported child lower-respiratory illness in the previous 12 months				
No	185 (94)	44 (97)	141 (94)	1.0
Yes	11 (6)	2 (4)	9 (6)	
Reported child diarrhea in the previous 12 months				
No	185 (94)	45 (98)	140 (93)	0.46
Yes	11 (6)	1 (2)	10 (7)	
Reported child vomited in the previous 12 months				
No	194 (99)	45 (98)	149 (99)	0.42
Yes	2 (1)	1 (2)	1 (< 1)	
Child consumed fish within previous 24 h				
No	173 (88)	37 (80)	136 (91)	0.04*
Yes	22 (11)	9 (20)	13 (9)	
Missing	1 (< 1)	0 (0)	1 (< 1)	
Child consumed tofu/soy milk within previous 24 h				
No	178 (91)	41 (89)	137 (91)	0.56
Yes	17 (9)	5 (11)	12 (8)	
Missing	1 (< 1)	0 (0)	1 (< 1)	
Child consumed cow’s milk within previous 24 h				
No	164 (84)	35 (78)	129 (86)	0.09
Yes	31 (16)	11 (24)	20 (13)	
Missing	1 (< 1)	0 (0)	1 (< 1)	
Child consumed eggs within previous 24 h				
No	164 (84)	41 (89)	123 (82)	0.36
Yes	31 (16)	5 (11)	26 (17)	
Missing	1 (< 1)	0 (0)	1 (< 1)	
Child consumed vegetables within previous 24 h				
No	22 (11)	4 (9)	18 (12)	0.61
Yes	173 (88)	42 (91)	131 (87)	
Missing	1 (< 1)	0 (0)	1 (< 1)	
Child consumed fruit within previous 24 h				
No	174	40 (87)	134 (89)	0.59
Yes	21	6 (13)	15 (10)	
Missing	1 (< 1)	0 (0)	1 (1)	
Child consumed pork within previous 24 h				
No	37 (19)	10 (22)	27 (18)	0.57
Yes	159 (81)	36 (78)	123 (82)	
Child consumed other meat within previous 24 h^f^				
No	150 (77)	37 (80)	113 (75)	0.52
Yes	45 (23)	99 (20)	36 (24)	
Missing	1 (< 1)	0 (0)	1 (< 1)	
Child consumed no meat within previous 24 h				
No	176 (90)	41 (89)	135 (90)	0.79
Yes	20 (10)	5 (11)	15 (10)	
Child consumed rice or rice porridge within previous 24 h				
No	0 (0)	0 (0)	0 (0)	NA
Yes	196 (100)	46 (100)	150 (100)	
Weight-for-age (z-score)	− 0.65 (− 2.8, 2.51)	− 0.825 (− 2.44, 1.61)	− 0.645 (− 2.8, 2.51)	0.79
Height-for-age (z-score)	− 0.62 (− 2.46, 1.79)	− 0.57 (− 2.43, 1.32)	− 0.73 (− 2.46, 1.79)	0.64
Weight-for-height (z-score)	− 0.36 (− 2.4, 3.54)	− 0.40 (− 2.14, 2.38)	− 0.35 (− 2.4, 3.54)	0.89
Bayley MDI	86 (62, 106)	90.5 (71, 104)	84 (62, 106)	0.0007***
Bayley PDI	92 (71, 122)	90 (75, 116)	92 (71, 122)	0.31

### Neurodevelopmental assessment

All 46 children were evaluated using the Bayley Scales within a nine-week window (child's age range: 36.0–37.9 months). Mean (± 1 SD) standardized MDI and Psychomotor Developmental Scores (PDI) scores were 90 ± 9.0 and 95 ± 13, respectively, which were slightly lower than the U.S. English-speaking standardization population (mean ± 1 SD: 100 ± 15)^12^. Lower MDI and/or PDI scores have been observed among other non-English speaking cohorts in Nepal^13^, Hokkaido, Japan^14^, and the Seychelles^15^.

### Children's gut microbiota coabundance scores

Table 2 includes bacterial coabundance groupings for the 25 most abundant gut microorganisms, using a 3-factor solution. The first factor captured coabundances of the genera, *Faecalibacterium, Clostridium* cluster XIVa*, Gemmiger, Phascolarctobacterium, Alistipes, Oscillibacter,* and *Sutterella* (range for positive loadings: 0.58, 0.85). This coabundance factor was negatively correlated with *Blautia, Anaerostipes, Clostridium* cluster XVIII, and *Streptococcus* (range for negative loadings: − 0.78, − 0.66). The second factor captured coabundances of *Blautia, Roseburia, Ruminococcus, Collinsella, Cellulosibacter*, *Coprococcus* (range for positive loadings: 0.45, 0.87), while *Bacteroides* and *Parabacteroides* were negatively correlated with this coabundance factor (range for negative loadings: − 0.77, − 0.61). The third factor captured abundances of the genera, *Faecalibacterium, Roseburia, Lachnospiraceae incertae sedis*, *Fusicatenibacter* and *Butyricicoccus* (range for positive loadings: 0.44, 0.64), while *Bifidobacterium*, *Ruminococcus,* and *Megamonas* were negatively correlated with this coabundance factor (range for negative loadings: − 0.58, − 0.42).

**Table 2:** Bacterial coabundance factor loadings for the 25 most abundant gut microorganisms (n = 46 children). Factor loadings > |0.40| are bolded.

Genus	Factor 1	Factor 2	Factor 3
*Bacteroides*	0.26	− **0.77**	− 0.33
*Faecalibacterium*	**0.59**	− 0.08	**0.44**
*Bifidobacterium*	− 0.43	0.17	− 0.58
*Blautia*	− **0.66**	**0.48**	0.05
*Roseburia*	0.19	**0.45**	**0.52**
*Clostridium XIVa*	**0.59**	− 0.24	0.37
*Lachnospiraceae incertae sedis*	− 0.18	0.16	**0.49**
*Gemmiger*	**0.58**	0.26	− 0.23
*Ruminococcus*	0.03	**0.51**	− **0.56**
*Parabacteroides*	0.08	− **0.61**	0.04
*Phascolarctobacterium*	**0.61**	− 0.31	− 0.15
*Anaerostipes*	− **0.78**	− 0.12	0.04
*Clostridium XVIII*	− **0.71**	− 0.09	0.40
*Alistipes*	**0.79**	0.13	− 0.07
*Megamonas*	− 0.25	0.23	− **0.42**
*Streptococcus*	− **0.67**	− 0.21	0.04
*Fusicatenibacter*	0.05	0.13	**0.64**
*Flavonifractor*	0.09	− 0.21	0.11
*Collinsella*	− 0.15	**0.87**	− 0.33
*Butyricicoccus*	− 0.37	0.33	**0.64**
*Oscillibacter*	**0.88**	− 0.13	0.27
*Cellulosibacter*	0.08	**0.85**	0.17
*Coprococcus*	0.10	**0.82**	− 0.13
*Escherichia shigella*	− 0.30	0.16	0.37
*Sutterella*	**0.85**	− 0.25	0.13

### Correlation between children's gut microbiota and maternal/child characteristics

For coabundance factor 1, children born by Cesarean birth (C-section) had lower values, compared to children born vaginally, while no differences were observed in alpha diversity measures (Supplementary Tables S1, S2). Neonatal gut microbiota differ between children born vaginally and by C-section^16^; however, the long-term impacts vary^17,18^. This may be due to other factors that impact children's later gut microbiota composition (e.g., infant feeding practices and child's diet)^5^.

For coabundance factor 2, children consuming fish within the previous 24 h had higher values, compared to children who did not (Supplementary Table S1). Coabundance factor 2 captured positive loadings for the genera *Roseburia, Coprococcus,* and *Blautia* (Table 2). Fish tissue is enriched in omega-3 fatty acids, including docosahexaenoic acid (DHA)^19^, which has been associated with enrichment of these taxa or taxa from the same family (*Lachnospiraceae*)^20,21^.

The second and third coabundance factors and/or alpha diversity indices were lower among children who had illnesses in the previous 12 months, including fever and respiratory illness, compared to children who did not (Supplementary Tables S1, S2), which was consistent with prior studies concerning childhood illnesses and reduced gut microbiota diversity^4,5^. The third co-abundance factor was also positively correlated with both gestational age and higher household income (Supplementary Tables S1 and S3), although the latter two variables were not correlated to each other (Wilcoxon rank sum test, p = 0.73).

### Multivariable regression models using coabundance factors

In both MDI and PDI models, the first coabundance factor was positively correlated with both outcome measures (Table 3). In adjusted models, for an interquartile range increase in the first coabundance factor 1 (loading: 2251), MDI scores increased on average by 3.9 points [95% Confidence Interval (CI): 0, 7.7], while PDI scores increased on average by 8.6 points (95% CI 3.1, 14). Compared to other covariates, the first coabundance factor explained the largest proportion of the variance in PDI scores (24%), while in the MDI model, the first coabundance factor explained 12% of the variance. In contrast, in the MDI and PDI models, the second coabundance factor explained < 1% and 9% of the variance, respectively, while the third coabundance factor explained 7% and 1% of the variance, respectively.

**Table 3:** Multivariable linear regression models relating the Bayley Scales of Infant Development-II to children's gut microbiota coabundance factors (n = 46 children). ^a^P-values are for the Beta coefficient: *p ≤ 0.05, **p < 0.01. ^b^For coabundance factors 1, 2, and 3, beta coefficients and 95% confidence intervals were multiplied by the interquartile range, of 2251, 3256, and 2516, respectively. ^c^Illnesses or symptoms included upper respiratory illness, lower respiratory illness, diarrhea, vomiting, or fever.

	Mental Developmental Index (MDI) r-squared = 0.55		Psychomotor Developmental Index (PDI) r-squared = 0.55	
	Beta (95% Confidence Interval)	Beta p-value^a^	Beta (95% Confidence Interval)	Beta p-value^a^
Coabundance Factor 1^b^ (unitless)	3.9 (0, 7.7)	0.049*	8.6 (3.1, 14)	0.003**
Coabundance Factor 2^b^ (unitless)	0.49 (− 4.7, 5.7)	0.85	6.6 (− 0.76, 14)	0.08
Coabundance Factor 3^b^ (unitless)	3.5 (− 1.1, 8.1)	0.13	− 2.0 (− 8.5, 4.6)	0.55
Mother's age (years)	− 0.28 (− 0.65, 0.09)	0.14	− 0.46 (− 0.99, 0.07)	0.09
Mother completed high school (yes)	3.6 (− 2.6, 9.8)	0.25	− 0.70 (− 9.5, 8.1)	0.87
Breastfeeding duration (months)	0.80 (− 0.01, 1.6)	0.053	− 0.45 (− 1.6, 0.71)	0.44
Child sex (male)	0.36 (− 4.9, 5.6)	0.89	− 11 (− 18, − 3.1)	0.007**
Child's age (months)	− 1.2 (− 6.0, 3.6)	0.63	3.9 (− 2.9, 11)	0.26
Child consumed fish within previous 24 h (yes)	5.0 (− 1.6, 12)	0.14	12 (2.2, 21)	0.02*
Child's weight for age (z-score)	3.2 (0.76, 5.7)	0.01*	2.4 (− 1.1, 5.9)	0.17
Child attends preschool (yes)	4.6 (− 1.1, 10)	0.11	0.31 (− 7.7, 8.4)	0.94
Birth mode (Cesarean = yes)	0.85 (− 4.4, 6.1)	0.75	2.4 (− 5.1, 9.8)	0.52
Child illness in the previous 12 months (yes)^c^	1.9 (− 4.1, 7.9)	0.52	3.7 (− 4.8, 12)	0.38

Child's weight-for-age (z-score) was associated with higher MDI scores (β = 3.2 points, 95% CI: 0.76, 5.7), while each 1-month increase in breastfeeding was associated with a 0.8-point (95% CI: − 0.01, 1.6) increase in the MDI score (Table 3). In contrast, the strongest predictors of PDI scores were child sex, with boys having lower scores compared to girls (β = − 11 points, 95% CI: − 18, − 3.1), while child consumption of fish in the previous 24 h was associated with higher PDI scores (β = 12 points, 95% CI: 2.2, 21) (Table 3).

### Multivariable regression models using alpha diversity measures and Maaslin2

Alpha diversity measures were not strongly correlated with Bayley MDI and PDI scores in bivariate analyses (Supplementary Table S3), and in adjusted regression models (Supplementary Table S4).

Using Maaslin2, in adjusted models, several taxa were correlated with Bayley MDI and PDI scores, although q-values were > 0.25 (Supplementary Table S5). The genus *Faecalibacterium* was positively correlated with both outcome measures, while the genus *Gemmiger* was positively correlated with PDI scores. Both taxa were among the dominant genera in the first coabundance factor (positive loadings: 0.59 and 0.58, respectively) (Table 2), which was also positively correlated with both MDI and PDI scored in adjusted models (Table 3). The genus *Lachnospiraceae incertae sedis* was negatively correlated with MDI scores, while the genus *Flavonifractor* was positively correlated with MDI scores (Supplementary Table S5). For both genera, the same directions of association were observed with coabundance factor 1 (negative and positive, respectively), although they were not among the dominant genera (loadings: -0.18 and 0.09, respectively) (Table 2).

### Sensitivity analyses

Models were re-evaluated, after replacing the standardized Bayley scores with the raw Bayley scores (Supplementary Table S6), and after excluding one child born pre-term (35.3 weeks) (Supplementary Table S7). For both analyses, the same patterns of association between Bayley scores and coabundance factors were observed as in our main analysis (Table 3).

## Discussion

At 36 months of age, children's gut microbiota composition was associated with Bayley scores. In adjusted models, the first bacterial coabundance factor (dominated by positive loadings for the genera *Sutterella, Oscillibacter*, and *Alistipes*, and negative loadings for the genera *Anaerostipes*, *Clostridium* cluster XVIII, and *Blautia*) was positively correlated with both MDI and PDI scores (Table 3), and explained a relatively large proportion of the variability in these scores in adjusted models (12% and 24%, respectively). Our results highlight the potential for microbial compositional characteristics to be important correlates of Bayley Scales performance in our small cross sectional assessment.

Findings in our study differed from previous studies investigating children's gut microbiota composition and neurodevelopment^6–8^. In North Carolina, USA, children's gut microbiota were clustered into three groups; the cluster represented by *Faecalibacterium* had the lowest cognitive scores (assessed using the Mullen Scales of Early Learning), while measures of alpha diversity (observed number of species, Chao1, Faith's phylogenetic diversity, and the Shannon index) were also associated with lower cognitive scores^7^. In our study, *Faecalibacterium* was associated with higher MDI and PDI scores (Table 3, Supplementary Table S4), while alpha diversity indices were not strongly correlated with MDI and PDI scores (Supplementary Table S3). In Massachusetts, Missouri, and California, USA, the bacterial coabundance factor with positive loadings for *Lachnospiraceae* was associated with poorer communication and personal and social skills in 3-year-olds (assessed using the Ages and Stages Questionnaire, 3rd edition)^6^. This differed from our study, in which the first coabundance factor contained both positive and negative loadings for genera of the *Lachnospiraceae* family, and this coabundance factor was correlated with better MDI and PDI scores (Table 3). Likewise, using Maaslin2, members from the *Lachnospiraceae* family (*Lachnospiraceae incertae sedis* and *Clostridium* cluster XlVb) were associated with both lower and higher MDI scores, respectively (Supplementary Table S5). In Ohio, USA, alpha diversity (phylogenetic diversity and the Shannon index) was associated with children's temperament, which was assessed by maternal report using the Early Childhood Behavior Questionnaire^8^. As noted above, Bayley Scales and alpha diversity indices were not strongly correlated in our study (Supplementary Tables S3, S4). Differences between our findings and results from other studies may reflect temporal development of the children's microbiota^5^, as well as potential differential sensitivity to the microbiome during different periods of neurodevelopment. In prior studies, children's gut microbiota were assessed at 6 months^6^, 1 year^7^, and 2 years^8^, while in our cross-sectional study, children's gut microbiota were assessed at 36–37 months, which is considered a stable phase in microbiome progression^5^.

One mechanism by which the gut microbiota is hypothesized to influence the central nervous system, is through the production of metabolites, including short-chain fatty acids (SCFAs)^22,23^. SCFAs enhance the integrity of the intestinal epithelial layer^24^, and also travel to other organs, including the brain^22,25^. In germ-free mice, the lack of a normal gut microbiota was associated with increased permeability of the blood–brain barrier^22^. Moreover, colonization of germ free mice by bacterial strains that produced SCFAs, including butyrate or acetate/propionate, reversed this trend and decreased permeability of the blood–brain barrier^22^.

Coabundance factor 1 captured positive loadings for the genus *Faecalibacterium* (loading: 0.59), which is considered one of the dominant butyrate producers and has been linked to anti-inflammatory effects^26^. Coabundance factor 1 also captured positive loadings for the genus *Clostridium* cluster XIVa (loading: 0.59), which includes a large number of butyrate-producing bacteria^27,28^, and positive loadings for the genus *Alistipes* (loading: 0.79), a propionate-producer^29^. *Blautia* spp. produce propionate^29^; however, this genus was negatively correlated with coabundance factor 1 (loading: − 0.66). Using Maaslin2, several butyrate- and propionate-producing bacteria were positively correlated with MDI and/or PDI scores, including *Faecalibacterium*^26^, members of *Clostridium* cluster XIVb^30^, *Butyricimonas*^31^, and *Flavonifractor*^29^ (Supplementary Table S4).

These results suggest the production of the SCFAs, including butyrate and/or propionate, may contribute positively to children's Bayley scores in our cohort. However, as noted above, this was not consistent across all components of coabundance factor 1. Furthermore, coabundance factor 2 captured positive loadings for *Roseburia, Coprococcus*, and *Blautia* (range for positive loadings: 0.45, 0.82) (Table 2), which are also known to produce butyrate and/or propionate^29^, and coabundance factor 3 captured positive loadings for the genera *Roseburia* and *Butyricicoccus* (loadings: 0.52, 0.64), which are both known to produce butyrate^29^. But coabundance factors 2 or 3 were not associated with Bayley scores (Table 3 and Supplementary Table S3), suggesting the contributions of butyrate and/or propionate production to associations were uncertain, and other factors likely contributed.

The highest positive loadings for coabundance factor 1 were for *Alistipes*, *Sutterella,* and *Oscillibacter* (positive loadings: 0.79, 0.85, and 0.88, respectively). In other studies, the same genera were adversely associated with cognitive outcomes^32–35^, which differed from the direction of association observed in our study. *Sutterella* spp. are considered a common intestinal commensal among adults and children, which decrease in abundance between the small intestine and the colon^36–38^. Studies concerning the role of *Sutterella* among children have focused on autism spectrum disorder (ASD). Compared to controls, among children with ASD, *Sutterella* spp. were higher in fecal samples^34^, and in ileal and cecal biopsies^35^. Similarly, clades within the genera *Oscillibacter* and *Alistipes* were enriched among adults with depression, compared to healthy controls^32,33^. *Oscillibacter* may contribute to depression due to production of valeric acid, which resembles the neurotransmitter GABA (gamma-aminobutyric acid)^33^. Disruption in GABA signaling has been linked to neurodevelopmental disorders^39^. Thus, in previous studies, all three genera were associated with poorer neurobehavioral function (childhood ASD and adult depression)^32–35^, whereas, in our analysis, they were associated with better neurocognitive function, i.e., higher MDI and PDI scores in association with increasing values for coabundance factor 1 (Table 3). This suggests the roles of these taxa may potentially vary by population characteristics (e.g., age, neurologic status) or by timing of microbiota assessment, and have yet to be elucidated. Lastly, in a cross-sectional analysis, there is the potential for reverse causation which may lead to divergent findings across studies, depending on how (or if) the outcome of interest impacts microbiota characteristics.

Although our study has many strengths, there are some limitations to note. Our sample size was limited; however, this study was exploratory. Despite the small sample size, our results suggest that gut ecology is an important correlate of children's neurodevelopment in our cohort. There were some differences between participants who collected their child's stool sample and those that did not (Table 1), which could have contributed to selection bias. The possibility of residual or unmeasured confounding remains, although we adjusted for many potential confounders. For example, we did not have information on potentially relevant factors, such as antibiotic treatment, which is known to impact the gut microbiota^4^. This was a cross-sectional study, and therefore it was not possible to determine the direction of association between children's gut microbiota and outcome measures. In addition, child's stool collection (at 3 years of age) occurred when microbiome development is considered more stable^5^. One potential ramification of measuring the microbiome when it is relatively stable is the underestimation of previous variation in the microbiome that could have impacted neurodevelopment. This is a form of measurement error and, as it is likely to be non-differential error, it would bias findings to the null. Thus, this timing of gut microbial assessment may lead to an underestimation of the “true” association. Longitudinal studies are needed to investigate whether there is a developmental window for the gut microbiome, which is most predictive for children's neurodevelopment.

In conclusion, in our cross sectional analysis of 36-month old children living in rural China, children's gut microbiota explained a large portion of variability in Bayley Scales (MDI and PDI scores). Results from this study suggest that gut microbiota may play an important role in children's neurodevelopment among typically developing children.

## Methods

### Recruitment

The study participants were residents of Daxin County, Guangxi province, China, which is predominantly rural, with a population of 359,800 (http://www.daxin.gov.cn/). Approximately 50,000 residents live in the small rural town of Daxin, and most of the remaining are rice farmers and live outside of town.

Between May 2013 and March 2014, 408 mother-infant pairs were recruited at the Maternal and Child Health Hospital in Daxin County. Eligible mothers were in good general health, resided in Daxin County during the previous 3 months, and planned to remain in Daxin County for the next 12 months. Mothers were enrolled up to 4 weeks before delivery or within one week postpartum. Of 408 mothers enrolled in the study, 10 mothers were subsequently excluded because they actually lived outside Daxin County (n = 3), gave birth to twins (n = 1), or data collection was incomplete (n = 6), resulting in a cohort of 398 mother-infant pairs. Participants returned between May 2016 and March 2017 for their child's 36-month assessment.

The research protocol was reviewed and approved by the Institutional Review Boards at Oregon State University (USA), the University of South Carolina (USA), and Xinhua Hospital (China). Mothers (or the child’s primary caregiver) provided written informed consent prior to their (and their child’s) participation. All methods were carried out in accordance with relevant guidelines and regulations.

### Questionnaires

After enrollment, while in the hospital, mothers filled out a detailed questionnaire ascertaining demographics, education, occupation, household income, pre-pregnancy weight and height (for body mass index calculation), delivery mode, and their child's sex. Gestational age was abstracted from the medical record based on maternal reported date of the last menstrual period and date of delivery. At 36 months, parents or caregivers completed a questionnaire regarding their child's health, breastfeeding duration, preschool attendance, primary caregiver, and their child's food intake during the previous 24 h. To characterize the child's health, caregivers were also asked whether the child had any of the following seven illnesses or symptoms in the previous 12 months: upper respiratory illness, lower respiratory illness, difficulty breathing, diarrhea, vomiting, fever, and rash; the frequency with which the illness or symptom occurred; and whether the child saw a doctor for the given illness or symptom. Children's weight and height were measured by hospital staff using a digital standing scale/stadiometer (Model # HCS-100-RT, Jiangsu, China). Z-scores for weight-for-age, height-for-age, and weight-for-height were calculated using the 2006 World Health Organization (WHO) child growth standards^40^, with the R package "anthro" (21 May 2020). Weight-for-age (z-score) reflects short-term nutritional deficiencies, while long-term deficiencies reduce stature, which are reflected in height-for-age and weight-for-height z-scores^41^.

### Neurodevelopmental assessment

At 36 months, children's neurodevelopment was assessed using the BSID-II, which was translated into Chinese. The BSID-II yields two main indices of performance: the Mental Developmental Index (MDI) and the Psychomotor Developmental Index (PDI)^12^. These indices are age-standardized to a mean of 100 and standard deviation of 15, based on an English language-speaking U.S. reference population^12^. The BSID-II underwent extensive pre-testing, and all children were evaluated by one doctor (co-author YN), who spoke the local dialect. The evaluator was trained on-site in Bayley administration by co-author EPT, a developmental psychologist. Examiner reliability was assessed throughout the follow-up period by videotaping five children (of 196 children = 2.6%). Videotapes for both MDI and PDI sections were evaluated and re-scored by co-authors EPT and FJB (also a developmental psychologist), and differences in scoring were minor.

### Analysis of children's gut microbiota

Participants collected their child's stool sample at home using a sterile cotton swab (Becton Dickinson Cat #220135, Franklin Lakes, NJ). The cotton swab was removed from the tube, and then used to swipe toilet paper containing the child's feces. The cotton swab was re-inserted into the tube, and within 24 h the stool sample was returned to the Maternal and Child Health Hospital in Daxin County and frozen (− 26 °C). Every 3–4 months, stool samples were transferred (by co-author SER) on dry ice or in a Credo Cube (− 20 °C) (Pelican BioThermal, Plymouth, MN, USA) from the hospital in Daxin County to Guangxi Medical University (in Nanning), and stored at − 80 °C.

In May 2017, all stool samples were transported on dry ice from Guangxi Medical University to the State Key Laboratory of Microbial Metabolism at Shanghai Jiao Tong University, and stored at − 80 °C until analysis. DNA extraction of all fecal samples was performed as previously described^42^. The concentration of extracted DNA was determined using a BioDrop (Biochrom Ltd., Cambridge, UK); its integrity and size was checked by 0.8% agarose gel electrophoresis containing 0.5 mg/ml ethidium bromide. Hypervariable region V3–V4 amplicons from the 16S rRNA gene were sequenced by Illumina MiSeq 2 × 300 bp paired-end sequencing (https://support.illumina.com/documents/documentation/chemistry_documentation/16s/16s-metagenomic-library-prep-guide-15044223-b.pdf). Detailed methods are in the Supplementary Information.

Quality-filtered reads were delineated into unique sequences and then sorted by decreasing abundance, and singletons were discarded. Using previously described methods^43^, operational taxa units (OTUs) were clustered de novo with Uparse at 97% similarity level^44^. Reference-based chimera detection was performed using UCHIME^45^ against the RDP (Ribosomal Database Project) classifier training database (v9)^46^. The OTU table was finalized by mapping quality-filtered reads to the remaining OTUs with the Usearch^47^ global alignment algorithm at a 97% cutoff. Sequence data were rarefied to 13,000 reads per sample (1000 permutations) to avoid bias caused by the difference in sequencing depth. Alpha diversity (within person diversity) was assessed using the number of observed OTUs, Shannon's diversity index, Faith's Phylogenetic Diversity, and Pielou's measure of evenness.

### Coabundance groupings

Coabundance groupings (or factor representations) of bacterial taxa within the children's gut were computed, similar to previously described methods^6^. Briefly, Spearman's rank correlation coefficients were determined for the 25 most abundant taxa (based on the median relative abundance), using the Compositionality Corrected by Permutation and Renormalization ("ccrepe") package in R^48^. Spearman's correlation matrix was used for principal components analysis (with varimax rotation), using the "psych" package in R^49^. This analysis revealed a 3-factor solution based on a scree plot. Factor loadings were applied to 16S rRNA sequencing counts to compute an individual's factor score, for each bacterial coabundance group.

### Data analysis

Differences in maternal/child characteristics between participants who provided a stool sample and those who did not were investigated using a Chi-squared test, Fisher's exact test, or Wilcoxon rank sum test. Bivariate associations between Bayley scores, children's gut microbiota (coabundance scores and alpha diversity measures), and maternal/child characteristics were then investigated using a Spearman's correlation, Wilcoxon rank sum test, or Kruskal–Wallis test. For bivariate associations and multivariable regression models, the number of levels for some categorical variables were combined: mothers completing high school and university were combined into one level (maternal education: < high school, ≥ high school), mothers who were workers (civil servant, white-collar worker, skilled worker, unskilled worker, and shopkeeper) or listed "other" as the type of work were combined into one category for non-farmers (maternal occupation: farmer, non-farmer), households earning 2000–5000 RMB per month and ≥ 5000 RMB per month were combined into one level (household monthly income: < 2000 RMB, ≥ 2000 RMB), and children cared for by their mothers or fathers were combined into one level, and were compared to children cared for by a grandparent (child's primary caregiver: mother/father, or grandparent).

Multivariable linear regression models were constructed to examine the relationships between Bayley scores (MDI and PDI) and children's gut microbiota. Primary analyses included all three coabundance factors as independent predictors. Table 1 includes all potential covariates that were available and considered for models based on evidence that they were independently associated with children's neurodevelopment and/or the gut microbiota in previous studies^4–11,15–21^. From this list, we developed a core model based on priors that included child's sex, child's age at BSID-II assessment, breastfeeding duration, and maternal age and education. The subset of additional Table 1 covariates that were included in the final models were chosen based on at least one of the following: their relationship with outcome measures; their relationship with coabundance factors; added variable plots; evidence of confounding of effect estimates for coabundance factors; and comparison of the Akaike Information Criterion between models with/without a given covariate^50^. From Table 1, child's rice consumption was the only variable not considered for inclusion because all children ingested rice or rice porridge in the previous 24 h.

Associations between Bayley scores and gut microbiota may differ between individual taxa and coabundance factors. Therefore, secondary analyses investigated the associations between covariate-adjusted Bayley scores (modeled as residuals) and individual taxa (genus-level), using Microbiome Multivariable Association with Linear Models (Maaslin2) in R^51^. For Maaslin2, individual taxa were included if measured in at least 20% of children's gut microbiota. Bacterial taxa were transformed using the default transformation in Maaslin2 (arcsine-square-root transformation), which has been applied to proportional data^52^. Secondary analyses also investigated the associations between Bayley scores and each alpha diversity measure in multivariable regression models.

Among the participants included in this analysis, just one variable (household income) had missing values, and missing data were imputed using multiple imputation based on the multivariate normal distribution^53^, as previously described^10^. Associations were checked with and without imputed data, and no differences were observed. As a sensitivity analysis, regression models using coabundance factors were re-evaluated using the Bayley raw scores in place of the age-standardized scores. One child was born pre-term (35.3 weeks), and as a sensitivity analysis, models using coabundance factors were re-evaluated including only children with term births (≥ 37 weeks). Assumptions for model residuals were checked (no evidence of non-linearity, constant variance, normal distribution) using appropriate plots. Potential influential observations were checked using Cook's distance; however none were observed.

For bivariate analyses and regression models, an alpha level of 0.05 was used as a guide for significance. To minimize the false discovery rate (FDR), we used dimensionality reduction (bacterial coabundance groups) in our primary analyses to characterize the gut microbiota, and thus q-values were not calculated. Using Maaslin2, q-values (FDR-corrected p-values) were calculated^46^, and q ≤ 0.25 was used as a guide for significance. Statistical analyses were performed using Stata (Version 9.2, College Station, TX, USA), and the R-platform (Version 4.0.2, 06 June 2020).

## Supplementary Information

Supplementary Information.

## Data Availability

The raw Illumina sequencing data generated in this study have been submitted to the Sequence Read Archive (SRA) at the National Center for Biotechnology Information (NCBI) under accession no. SRP302243.
