# Supplementary material for: Neurodevelopment correlates with gut microbiota in a cross-sectional analysis of children at 3 years of age in rural China
Source: Sci Rep. 2021 Apr 1;11:7384. doi: 10.1038/s41598-021-86761-7 (PMC8016964; doi:10.1038/s41598-021-86761-7)
Supplement: Supplementary file 1 — Supplementary Information. [file 41598_2021_86761_MOESM1_ESM.pdf]

Supplementary Information: NEURODEVELOPMENT CORRELATES WITH GUT MICROBIOTA IN A CROSS-SECTIONAL ANALYSIS OF CHILDREN AT THREE YEARS OF AGE IN RURAL CHINA

Sarah E. Rothenberg, Qiurong Chen, Jian Shen, Yanfen Nong, Hua Nong, Eva P. Trinh, Fred J. Biasini, Jihong Liu, Xiaoyun Zeng, Yunfeng Zou, Fengxiu Ouyang, Susan A. Korrick

Table of Contents

Detailed methods for analysis of children's gut microbiota	pg. 2
Table S1	pg. 3-6
Table S2	pg. 7-10
Table S3	pg. 11-12
Table S4	pg. 13
Table S5	pg. 14
Table S6	pg. 15
Table S7	pg. 16
References	pg. 17

Detailed methods for analysis of children's gut microbiota

The primers used for amplicon PCR were S-D-Bact-0341-b-S-17, 5’-CCTACGGGNGGCWGCAG-3’ and S-D-Bact-0785-a-A-21, 5’-GACTACHVGGGTATCTAATCC-3’ (Klindworth et al., 2013). For the amplicon PCR (amplification of 16S rRNA gene V3-V4 region), the 25 μl reaction mix consisted of 1 × Phanta Max Buffer, 0.2 mM dNTP, 0.2 μM of each specific primer for V3-V4 region of 16S rRNA gene, 0.5 U of Phanta Max Super-Fidelity DNA Polymerase (P505, Vazyme,Nanjing), and 20 ng template DNA. The PCR cycle number was reduced to 21 to diminish bias. The program was started with pre-denaturation at 95°C for 3 min, followed by 21 cycles of denaturation at 95°C for 30 s, annealing at 55°C for 30 s and extension at 72°C for 30 s, and ended up with a final extension at 72°C for 5 min. For the Index PCR (attachment of dual indices and Illumina sequencing adapter using the Nextera XT Index Kit), the 25 μl reaction mix consisted of 1 × Phanta Max Buffer, 0.2 mM dNTP, 2.5 μl of each N7 and S5 Index primers as described in the protocol, 0.5 U of Phanta Max Super-Fidelity DNA Polymerase, and 2.5 μl purified products of the Amplicon PCR step as template DNA. The PCR program of Index PCR was the same as Amplicon PCR except that the cycle number was reduced to 8. The purified products of the Index PCR were mixed at equal ratio and sequenced using the Illumina MiSeq System (Illumina Inc., San Diego, CA., USA).

Table S1. Distribution of Bayley Scales of Infant Development, 2nd Edition scores, and bacterial coabundance factor loadings, by maternal/child characteristics (n=46 children). [a]P-values are for Kruskal-Wallis test or Wilcoxon rank sum test: *$p \leq 0.05$, ** $p \leq 0.01$, *** $p < 0.001$, [b]BMI for Asian populations: underweight (BMI< 18.5 kg/m^2), normal weight (18.5 kg/m^2 ≤ BMI < 23 kg/m^2), overweight (23 kg/m^2 ≤ BMI < 27.5 kg/m^2), and obese (BMI ≥ 27.5 kg/m^2) (World Health Organization, 2004). [c]Non-farmers include categories for workers (civil servant, white-collar worker, skilled worker, unskilled worker, and shopkeeper), unemployed and other. [d]Illnesses queried included upper respiratory illness, lower respiratory illness, diarrhea, vomiting, and fever. [e]Other meat includes sheep, beef, chicken, or duck. Abbreviations: BMI (Body Mass Index), MDI (Mental Developmental Index), PDI (Psychomotor Developmental Index), RMB (ren min bi)

	**N (%)**	**MDI Median (range)**	**p-value[a]**	**PDI Median (range)**	**p-value[a]**	**Factor 1 Median (range)**	**p-value[a]**	**Factor 2 Median (range)**	**p-value[a]**	**Factor 3 Median (range)**	**p-value[a]**
**Questionnaire Responses upon Enrollment**											
**Mother's prepregnancy BMI[b]**											
Underweight	11 (24)	88 (71, 104)	0.35	88 (81, 116)	0.83	1240 (-2480, 4050)	0.96	-2240 (-4680, 2190)	0.46	-930 (-3520, 1490)	0.30
Normal weight	23 (50)	88 (72, 102)		88 (75, 116)		740 (-3630, 3050)		-869 (-4400, 3220)		-502 (-2980, 2350)	
Overweight or obese	12 (26)	92.5 (77, 102)		96 (75, 116)		981 (-3220, 3381)		-574 (-4640, 905)		89.7 (-2900, 2600)	
**Mother's education completed**											
< High School	37 (80)	89 (71, 102)	0.25	88 (75, 116)	0.45	715 (-3220, 4050)	0.45	-869 (-4680, 3220)	0.59	-50.9 (-3520, 2600)	0.09
≥ High School	9 (20)	98 (80, 104)		92 (82, 116)		1350 (-3630, 3820)		-1920 (-2950, 609)		-930 (-2900, 603)	
**Mother's occupation**											

Non-Farmer[c]	12 (26)	96 (77, 104)	0.21	88 (75, 116)	0.92	1310 (-3630, 3820)	0.25	-1520 (-4680, 905)	0.57	-557 (-2280, 1130)	0.65
Farmer	34 (74)	90 (71, 102)		92 (75, 116)		693 (-3220, 4050)		-887 (-4640, 3220)		-532 (-3520, 2600)	
**Household monthly income (RMB)**											
Income < 2000	25 (54)	90 (71, 102)	0.63	92 (79, 116)	0.29	600 (-3220, 3820)	0.16	-906 (-4680, 3220)	0.35	-1130 (-3520, 1750)	0.01**
Income ≥ 2000	21 (46)	91 (72, 104)		88 (75, 116)		1350 (-3630, 4050)		-1530 (-4640, 1730)		-603 (-2980, 2600)	
**Cesarean birth**											
No	34 (74)	90 (71, 104)	0.99	90 (76, 116)	0.93	1430 (-3220, 4050)	0.045*	-964 (-4680, 3220)	0.71	-532 (-3520, 2600)	0.75
Yes	12 (26)	91.5 (72, 102)		90 (75, 116)		574 (-3630, 1880)		-454 (-3300, 1940)		-582 (-2980, 1130)	
**Child sex**											
Male	28 (61)	91 (71, 102)	0.75	88 (75, 112)	0.007**	681 (-3220, 3820)	0.49	-895 (-4400, 3220)	0.67	-954 (-3520, 2600)	0.28
Female	18 (39)	90.5 (72, 104)		102.5 (82, 116)		1250 (-3630, 4050)		-1410 (-4680, 1940)		10.3 (-2900, 2340)	
**Questionnaire responses at 36 months**											
**Primary caregiver**											
Mother/Father	35 (76)	92 (71, 104)	0.29	92 (75, 116)	0.71	943 (-3630, 4050)	0.95	-869 (-4400, 3220)	0.08	-518 (-3520, 2600)	0.60
Grandparent	11 (24)	90 (72, 100)		88 (76, 116)		1240 (-2410, 2290)		-2490 (-4680, 2190)		-611 (-2860, 1670)	
**Child attends preschool**											
No	14 (30)	89 (72, 98)	0.13	88 (76, 116)	0.29	829 (-3220, 2410)	0.76	-745 (-4680, 1940)	0.91	-245 (-2370, 2600)	0.21
Yes	32 (70)	91.5 (71, 104)		92 (75, 116)		1140 (-3630, 4050)		-964 (-4640, 3220)		-827 (-3520, 2350)	
**Reported any child illness in the previous 12 months[d]**											
No	13 (28)	92 (72, 102)	0.76	92 (76, 116)	0.67	943 (-3630, 3820)	0.85	-288 (-2880, 1940)	0.16	603 (-1820, 2600)	0.02*
≥ 1 illness (range: 1-4)	33 (72)	89 (71, 104)		88 (75, 116)		1020 (-3220, 4050)		-1030 (-4680, 3220)		-725 (-3520, 2350)	

**Reported child fever in the previous 12 months**										
No	37 (80)	91 (71, 104)	0.71	92 (75, 116)	0.66	1020 (-3630, 4050)	0.86	-858 (-4400, 3220)	0.05*	-50.9 (-3520, 2600)	0.04*
Yes	9 (20)	88 (72, 102)		88 (75, 116)		740 (-1290, 2300)		-2300 (-4680, 430)		-1730 (-2980, 1750)	
**Reported child upper-respiratory illness in the previous 12 months**											
No	19 (41)	91 (71, 102)	0.69	88 (75, 116)	0.78	740 (-3630, 3820)	0.92	-529 (-3302, 1940)	0.43	-50.9 (-3520, 2600)	0.27
Yes	27 (59)	90 (72, 104)		92 (75, 116)		1240 (-3220, 4050)		-1030 (-4680, 3220)		-612 (-2900, 2350)	
**Child consumed fish within previous 24 hr**											
No	37 (80)	88 (71, 104)	0.011*	88 (75, 116)	0.07	1240 (-3630, 4050)	0.24	-1000 (-4680, 3220)	0.03*	-503 (-3520, 2600)	0.94
Yes	9 (20)	98 (80, 102)		108 (79, 116)		-66.5 (-3220, 3380)		609 (-2300, 2190)		-725 (-2280, 2210)	
**Child consumed tofu/soy milk within previous 24 hr**											
No	41 (89)	90 (71, 104)	0.87	92 (75, 116)	0.83	943 (-3630, 4050)	0.82	906 (-4640, 3220)	0.24	-664 (-3520, 2600)	0.45
Yes	5 (11)	92 (80, 98)		88 (88, 104)		1240 (-3220, 2400)		-2820 (-4680, 641)		-503 (-612, 758)	
**Child consumed cow's milk within previous 24 hr**											
No	35 (76)	91 (71, 104)	0.61	88 (75, 116)	0.43	715 (-3630, 4050)	0.18	-869 (-4680, 3220)	0.48	-545 (-3520, 2600)	0.71
Yes	11 (24)	90 (82, 102)		92 (75, 116)		1480 (-2410, 3380)		-1530 (-3970, 2190)		-439 (-2980, 1750)	
**Child consumption of eggs within previous 24 hr**											
No	41 (89)	91 (71, 104)	0.44	88 (75, 116)	0.96	943 (-3630. 4050)	0.82	-906 (-4680, 3220)	0.96	-503 (-3520, 2600)	0.24
Yes	5	84		92		1470		-1030		-1440	

	(11)	(77, 98)		(75, 112)		(-564, 1880)		(-3970, 2190)		(-2500, 1130)	
**Child consumption of vegetables within previous 24 hr**											
No	4 (9)	83 (72, 102)	0.58	86.5 (82, 92)	0.23	505 (-1290, 1350)	0.37	-1710 (-2880, -37.6)	0.64	-646 (-2370, 617)	0.70
Yes	42 (1)	90.5 (71, 104)		92 (75, 116)		1130 (-3630, 4050)		-913 (-4680, 3220)		-532 (-3520, 2600)	
**Child consumption of fruit within previous 24 hr**											
No	40 (87)	90.5 (71, 104)	0.33	90 (75, 116)	0.69	829 (-3220, 4050)	0.54	-887 (-4640, 3220)	0.36	-511 (-3520, 2600)	0.77
Yes	6 (13)	94 (86, 102)		90 (88, 116)		1290 (-3630, 3820)		-1870 (-4680, 430)		-668 (-1360, 1750)	
**Child consumption of pork within previous 24 hr**											
No	10 (22)	92.5 (72, 104)	0.90	98 (79, 116)	0.41	1090 (-3220, 4050)	0.77	-1890 (-3970, 641)	0.23	-479 (-2510, 1490)	0.98
Yes	36 (78)	90 (71, 102)		88 (75, 116)		981 (-3630, 3820)		-863 (-4680, 3220)		-578 (-3520, 2600)	
**Child consumption of other meat within previous 24 hr**[e]											
No	37 (80)	91 (71, 102)	0.96	92 (75, 116)	0.65	715 (-3220, 3820)	0.10	-858 (-4684, 3220)	0.26	-612 (-3520, 2600)	0.24
Yes	9 (20)	88 (72, 104)		88 (82, 116)		1840 (-3630, 4050)		-1530 (-3180, 52.1)		603 (-2280, 1490)	
**Child consumption of no meat within previous 24 hr**											
No	41 (89)	90 (71, 104)	0.67	88 (75, 116)	0.76	1240 (-3630, 4050)	0.19	-921 (-4680, 3220)	0.96	-545 (-3520, 2600)	0.56
Yes	5 (11)	91 (80, 98)		92 (79, 116)		-6.17 (-3220, 1480)		-621 (-3970, 641)		-518 (-2510, 617)	

Table S2. Distribution of alpha diversity measures by maternal/child characteristics (n=46 children). [a]P-values are for Kruskal-Wallis test or Wilcoxon rank sum test: $*p \leq 0.05$, $** p \leq 0.01$, $*** p < 0.001$, [b]BMI for Asian populations: underweight (BMI< 18.5 kg/m^2), normal weight (18.5 kg/m^2 ≤ BMI < 23 kg/m^2), overweight (23 kg/m^2 ≤ BMI < 27.5 kg/m^2), and obese (BMI ≥ 27.5 kg/m^2) (World Health Organization, 2004). [c]Non-farmers include categories for workers (civil servant, white-collar worker, skilled worker, unskilled worker, and shopkeeper), unemployed and other. [d]Illnesses queried included upper respiratory illness, lower respiratory illness, diarrhea, vomiting, and fever. [e]Other meat includes sheep, beef, chicken, or duck. Abbreviations: BMI (Body Mass Index), MDI (Mental Developmental Index), PDI (Psychomotor Developmental Index), RMB (ren min bi)

	N (%)	# OTUs Median (range)	p-value[a]	Shannon Median (range)	p-value[a]	Faith's PD Median (range)	p-value[a]	Pielou Median (range)	p-value[a]
**Questionnaire Responses upon Enrollment**									
**Mother's prepregnancy BMI[b]**									
Underweight	11 (24)	108 (65, 133)	0.30	4.8 (3.2, 5.8)	0.12	8.2 (6.4, 11)	0.59	0.73 (0.52, 0.82)	0.06
Normal weight	23 (50)	112 (54, 231)		5.0 (4.0, 6.2)		8.5 (5.2, 15)		0.74 (0.61, 0.80)	
Overweight or obese	12 (26)	118 (88, 209)		5.4 (4.5, 6.4)		9.1 (6.8, 14)		0.77 (0.70, 0.83)	
**Mother's education completed**									
< High School	37 (80)	116 (65, 231)	0.07	5.0 (3.2, 6.4)	0.27	8.5 (5.2, 15)	0.17	0.75 (0.52, 0.83)	0.63
≥ High School	9 (20)	88 (54, 133)		4.7 (4.2, 5.8)		7.2 (5.8, 11)		0.73 (0.65, 0.82)	
**Mother's occupation**									
Non-Farmer[c]	12 (26)	121 (54, 209)	0.94	5.3 (4.2, 6.4)	0.76	9.5 (5.8, 14)	0.55	0.76 (0.65, 0.83)	0.38
Farmer	34	110.5		5.0		8.4		0.75	

	(74)	(77, 231)		(3.2, 6.2)		(5.2, 15)		(0.52, 0.81)	
**Household monthly income (RMB)**									
Income < 2000	25 (54)	110 (54, 169)	0.47	5.0 (4.0, 5.9)	0.52	8.2 (5.2, 14)	0.67	0.74 (0.62, 0.81)	0.49
Income ≥ 2000	21 (46)	116 (75, 231)		5.1 (3.2, 6.4)		8.5 (6.7, 15)		0.76 (0.52, 0.83)	
**Cesarean birth**									
No	34 (74)	110.5 (54, 231)	0.74	4.9 (3.2, 6.2)	0.41	8.5 (5.2, 15)	0.98	7.5 (0.52, 0.82)	0.53
Yes	12 (26)	114 (85, 209)		5.1 (4.2, 6.4)		8.4 (6.8, 12)		0.76 (0.65, 0.83)	
**Child sex**									
Male	28 (61)	111 (54, 231)	0.38	5.0 (4.0, 6.4)	0.79	8.1 (5.2, 15)	0.46	0.75 (0.62, 0.83)	0.95
Female	18 (39)	119 (65, 169)		5.1 (3.2, 5.8)		8.8 (6.4, 14)		0.75 (0.52, 0.82)	
**Questionnaire responses at 36 months**									
**Primary caregiver**									
Mother/Father	35 (76)	116 (54, 231)	0.39	5.1 (3.2, 6.4)	0.69	8.9 (5.2, 15)	0.31	0.74 (0.52, 0.83)	0.71
Grandparent	11 (24)	103 (65, 143)		4.9 (4.4, 5.7)		8.1 (6.4, 10)		0.75 (0.68, 0.81)	
**Child attends preschool**									
No	14 (30)	123 (65, 231)	0.36	5.4 (4.4, 6.2)	0.21	8.7 (6.4, 15)	0.55	0.78 (0.68, 0.81)	0.053
Yes	32 (70)	110 (54, 209)		5.0 (3.2, 6.4)		8.4 (5.2, 14)		0.74 (0.52, 0.83)	
**Reported any child illness in the previous 12 months[d]**									
No	13 (28)	130 (85, 231)	0.003**	5.5 (4.2, 6.2)	0.01**	9.6 (7.2, 15)	0.006**	0.78 (0.65, 0.81)	0.06
≥ 1 illness (range: 1-4)	33 (72)	102 (54, 209)		4.9 (3.2, 6.4)		8.0 (5.2, 12)		0.74 (0.52, 0.83)	
**Reported child fever in the previous 12 months**									
No	37	116	0.03*	5.2	0.09	8.9	0.011*	0.75	0.27

	(80)	(54, 231)		(3.2, 6.4)		(5.2, 15)		(0.52, 0.83)	
Yes	9 (20)	91 (65, 126)		4.9 (4.4, 5.3)		7.5 (6.4, 8.2)		0.75 (0.68, 0.76)	
**Reported child upper-respiratory illness in the previous 12 months**									
No	19 (41)	126 (85, 231)	0.03*	5.3 (4.2, 6.2)	0.09	8.9 (6.8, 15)	0.01*	0.76 (0.65, 0.81)	0.27
Yes	27 (59)	100 (54, 209)		4.9 (3.2, 6.4)		8.1 (5.2, 12)		0.74 (0.52, 0.83)	
**Child consumed fish within previous 24 hr**									
No	37 (80)	111 (65, 231)	0.94	5.0 (3.2, 6.4)	0.73	8.5 (5.2, 15)	0.77	0.75 (0.52, 0.83)	0.67
Yes	9 (20)	116 (54, 169)		5.1 (4.2, 5.7)		8.2 (5.8, 14)		0.76 (0.73, 0.77)	
**Child consumed tofu/soy milk within previous 24 hr**									
No	41 (89)	116 (54, 209)	0.27	5.1 (3.2, 6.4)	0.54	8.5 (5.2, 14)	0.25	0.75 (0.52, 0.83)	0.99
Yes	5 (11)	88 (65, 231)		4.9 (4.5, 6.2)		6.8 (6.4, 15)		0.75 (0.72, 0.79)	
**Child consumed cow's milk within previous 24 hr**									
No	35 (76)	116 (54, 231)	0.88	5.0 (3.2, 6.4)	0.60	8.5 (5.2, 15)	0.77	0.75 (0.52, 0.83)	0.67
Yes	11 (24)	102 (87, 169)		5.1 (4.5, 5.9)		8.1 (6.8, 14)		0.75 (0.70, 0.81)	
**Child consumption of eggs within previous 24 hr**									
No	41 (89)	116 (65, 231)	0.45	0.51 (3.2, 6.2)	0.79	8.5 (5.2, 15)	0.47	0.75 (0.52, 0.82)	0.87
Yes	5 (11)	103 (54, 209)		5.0 (4.2, 6.4)		7.7 (5.8, 12)		0.75 (0.73, 0.83)	
**Child consumption of vegetables within**									

**previous 24 hr**									
No	4 (9)	99.5 (88, 126)	0.49	4.7 (4.4, 5.7)	0.37	7.9 (7.3, 9.7)	0.82	0.71 (0.68, 0.81)	0.31
Yes	42 (1)	114 (54, 231)		5.1 (3.2, 6.4)		8.5 (5.2, 15)		0.75 (0.52, 0.83)	
**Child consumption of fruit within previous 24 hr**									
No	40 (87)	116 (54, 231)	0.07	5.1 (3.2, 6.4)	0.22	8.6 (5.2, 15)	0.18	0.75 (0.52, 0.83)	0.56
Yes	6 (13)	93 (65, 128)		4.7 (4.2, 5.5)		7.3 (6.4, 11)		0.74 (0.65, 0.79)	
**Child consumption of pork within previous 24 hr**									
No	10 (22)	114 (54, 133)	0.41	5.1 (3.2, 5.8)	0.98	8.2 (5.8, 9.9)	0.37	0.76 (0.52, 0.82)	0.39
Yes	36 (78)	112 (65, 231)		5.0 (4.0, 6.4)		8.5 (5.2, 15)		0.74 (0.61, 0.83)	
**Child consumption of other meat within previous 24 hr[e]**									
No	37 (80)	111 (65, 209)	0.80	5.0 (4.0, 6.4)	0.67	8.5 (5.2, 14)	0.57	0.75 (0.61, 0.83)	0.38
Yes	9 (20)	119 (54, 231)		5.2 (3.2, 6.2)		8.9 (5.8, 15)		0.74 (0.52, 0.82)	
**Child consumption of no meat within previous 24 hr**									
No	41 (89)	111 (54, 231)	0.92	5.0 (3.2, 6.4)	0.41	8.5 (5.2, 15)	0.54	0.74 (0.52, 0.83)	0.09
Yes	5 (11)	126 (87, 130)		5.3 (4.8, 5.7)		8.2 (6.8, 9.7)		0.76 (0.75, 0.81)	

Table S3. Spearman's correlation matrix for maternal/child characteristics, Bayley Scales of Infant Development, 2nd Edition, bacterial coabundance factor loadings, and alpha diversity measures (n=46 children). P-values are for Spearman's correlation:*p < 0.05, ** p<0.01, *** p<0.001, Abbreviations: Faith's PD (Faith's Phylogenetic Diversity), HAZ (height-for-age z-score), MDI (Mental Developmental Index), OTU (Operational taxa units), PDI (Psychomotor Developmental Index), Pielou (Pielou's measure of evenness), WAZ (weight-for-age z-score), WHZ (weight-for-height z-score)

	**MDI**	**PDI**	**Factor 1**	**Factor 2**	**Factor 3**	**# OTUs**	**Shan-non**	**Faith's PD**	**Pie-lou**	**Mother's age (years)**	**Gestational age (weeks)**	**Child's age (months)**	**Breast-feeding (months)**	**WAZ**	**HAZ**	**WHZ**
**MDI**	1															
**PDI**	**0.58*** **	1														
**Factor 1**	**0.29***	**0.31***	1													
**Factor 2**	-0.02	0.04	**-0.54*** **	1												
**Factor 3**	0.13	0.08	0.21	0.19	1											
**# OTUs**	0.04	-0.06	0.22	**0.30***	**0.34***	1										
**Shannon**	0.16	0.03	**0.31***	0.24	0.28	**0.90*** **	1									
**Faith's PD**	0.05	-0.08	0.22	**0.33***	**0.30***	**0.93*** **	**0.82*** **	1								
**Pielou**	0.22	0.09	**0.31***	0.15	0.25	**0.69*** **	**0.91*** **	**0.59*** **	1							
**Mother's age (years)**	-0.13	**-0.32***	<0.01	<0.01	0.13	-0.08	-0.17	<0.01	-0.24	1						
**Gestational age (weeks)**	0.08	0.20	0.10	0.25	**0.35***	0.11	0.11	0.12	0.14	-0.08	1					
**Child's age (months)**	-0.13	0.10	-0.12	0.16	-0.09	<0.01	-0.08	<0.01	-0.14	<0.01	0.12	1				
**Breastfeeding (months)**	0.12	-0.06	-0.05	<0.01	-0.24	0.20	0.13	0.16	0.05	0.07	<0.01	0.03	1			
**WAZ**	**0.41****	0.21	-0.07	0.05	0.14	-0.25	-0.16	-0.23	-0.03	0.05	0.09	-0.16	-0.10	1		
**HAZ**	**0.39****	0.17	-0.07	0.14	0.23	-0.16	-0.16	-0.12	-0.11	0.07	0.05	-0.16	-0.05	**0.76*** **	1	

**WHZ**	0.25	0.09	-0.10	-0.02	0.04	-0.23	-0.10	-0.26	0.05	0.05	0.04	-0.11	-0.14	**0.86*** **	**0.38** **	1

Table S4. Four multivariable linear regression models evaluating the associations between Bayley Scales of Infant Development, 2nd Edition and each of the four alpha diversity measures (n=46 children). [a]P-values are for the Beta coefficients. [b]Each model was adjusted for mother's age (continuous), whether the mother completed high school (yes/no), breastfeeding duration (months), child sex, child's age (months), child fish consumption in the previous 24 hours (yes/no), child's weight-for-age (z-score), whether the child attends preschool (yes/no), birth mode (Cesarean = yes), and whether the child had an illness in the previous 12 months (yes/no). Illnesses included upper respiratory illness, lower respiratory illness, diarrhea, vomiting, or fever.

		**Mental Developmental Index**		**Psychomotor Developmental Index**	
	**Alpha diversity measures**	**Beta (95% Confidence Interval)**	**Beta p-value[a]**	**Beta (95% Confidence Interval)**	**Beta p-value[a]**
**Model 1[b]**	**# of Operational Taxa Units (OTUs)**	0.04 (-0.04, 0.13)	0.28	0.02 (-0.10, 0.14)	0.79
**Model 2[b]**	**Shannon**	2.4 (-2.0, 6.8)	0.27	1.1 (-5.3, 7.4)	0.74
**Model 3[b]**	**Faith's Phylogenetic Diversity**	1.0 (-0.42, 2.4)	0.16	0.61 (-1.4, 2.7)	0.55
**Model 4[b]**	**Pielou's Evenness**	21 (-25, 68)	0.35	13 (-53, 80)	0.69

Table S5. Results from adjusted regression models evaluating the associations between Bayley Scales of Infant Development, 2nd Edition and gut microbiota taxa, using Microbiome Multivariable Association with Linear Models (Maaslin2) (n=46 children). [a]The residuals of the Bayley Scores were used in the regression models, after adjustment for mother's age (continuous), whether the mother completed high school (yes/no), breastfeeding duration (months), child sex, child's age (months), child fish consumption in the previous 24 hours (yes/no), child's weight-for-age (z-score), whether the child attends preschool (yes/no), birth mode (Cesarean = yes), and whether the child had an illness in the previous 12 months (yes/no). Illnesses included upper respiratory illness, lower respiratory illness, diarrhea, vomiting, or fever. [b]P-values and q-values [false-discovery rate (FDR)-adjusted p-values] are for the Beta coefficients.

a. Mental Developmental Index (MDI)[a]

Genus	Beta Coefficient	Number of samples not 0	p-value[b]	q-value[b] (FDR adjusted p-value)
Lachnospiraceae incertae sedis	-0.0037	45	0.010	0.59
Butyricimonas	0.0016	27	0.017	0.59
Granulicatella	-0.00041	42	0.027	0.59
Flavonifractor	0.0016	46	0.028	0.59
Faecalibacterium	0.0058	46	0.043	0.59
Abiotrophia	-0.00021	14	0.046	0.59
Clostridium XlVb	0.0011	36	0.047	0.59

b. Psychomotor Developmental Index (PDI)[a]

Genus	Coefficient	Number of samples not 0	p-value[b]	q-value[b] (FDR adjusted p-value)
Faecalibacterium	0.0054	46	0.0051	0.33
Gemmiger	0.0043	44	0.0068	0.33
Butyricimonas	0.00099	27	0.029	0.95

Table S6. Multivariable linear regression models, relating the Bayley Scales of Infant Development, 2nd Edition (raw scores) to children's gut microbiota (coabundance factors) (n=46 children). [a]P-values are for the Beta coefficients: *p≤0.05, **p<0.01. [b]For coabundance factors 1, 2, and 3, beta coefficients and 95% confidence intervals were multiplied by the interquartile range of 2251, 3256, and 2516, respectively. [c]Illnesses included upper respiratory illness, lower respiratory illness, diarrhea, vomiting, or fever.

	**Mental Developmental Index (raw scores) r-squared = 0.51**		**Psychomotor Developmental Index (raw scores) r-squared = 0.55**	
	**Beta (95% Confidence Interval)**	**Beta p-value[a]**	**Beta (95% Confidence Interval)**	**Beta p-value[a]**
**Coabundance Factor 1[b] (unitless)**	2.0 (0.03, 4.0)	0.047*	2.3 (0.79, 3.8)	0.004**
**Coabundance Factor 2[b] (unitless)**	0.40 (-2.3, 3.1)	0.76	1.9 (-0.17, 3.9)	0.07
**Coabundance Factor 3[b] (unitless)**	1.8 (-0.57, 4.2)	0.13	-0.44 (-2.3, 1.4)	0.62
**Mother's age (years)**	-0.12 (-0.31, 0.07)	0.20	-0.11 (-0.26, 0.04)	0.20
**Mother completed high school (yes)**	1.9 (-1.3, 5.1)	0.23	-0.05 (-2.5, 2.4)	0.97
**Breastfeeding duration (months)**	0.36 (-0.06, 0.78)	0.09	-0.15 (-0.47, 0.17)	0.34
**Child sex (male)**	0.17 (-2.5, 2.9)	0.90	-2.8 (-4.9, -0.75)	0.009**
**Child's age (months)**	1.6 (-0.82, 4.1)	0.18	2.2 (0.29, 4.1)	0.03*
**Child consumed fish within previous 24 hour (yes)**	2.5 (-0.88,5.9)	0.14	2.9 (0.30, 5.5)	0.03*
**Child's weight for age (z-score)**	1.4 (0.14, 2.7)	0.03*	0.55 (-0.42, 1.5)	0.26
**Child attends preschool (yes)**	2.2 (-0.74, 5.1)	0.14	0.006 (-2.2, 2.2)	1.0
**Birth mode (Cesarean = yes)**	0.74 (-2.0, 3.4)	0.58	0.81 (-1.3, 2.9)	0.43
**Child illness in the previous 12 months (yes)[c]**	1.2 (-1.9, 4.3)	0.43	1.1 (-1.2, 3.5)	0.34

Table S7. Results for multivariable linear regression, relating the Bayley Scales of Infant Development, 2nd Edition, to children's gut microbiota (coabundance factors), including children with ≥37 weeks gestational age (n=45 children). [a]P-values are for the Beta coefficients: *p≤0.05, **p<0.01. [b]For coabundance factors 1, 2, and 3, beta coefficients and 95% confidence intervals were multiplied by the interquartile range of 2251, 3256, and 2516, respectively. [c]Illnesses included upper respiratory illness, lower respiratory illness, diarrhea, vomiting, or fever.

	**Mental Developmental Index r-squared = 0.59**		**Psychomotor Developmental Index r-squared = 0.54**	
	**Beta (95% Confidence Interval)**	**Beta p-value[a]**	**Beta (95% Confidence Interval)**	**Beta p-value[a]**
**Coabundance Factor 1[b] (unitless)**	4.2 (0.46, 7.9)	0.03*	8.6 (3.0, 14)	0.004**
**Coabundance Factor 2[b] (unitless)**	1.6 (-3.5, 6.7)	0.53	6.6 (-1.1, 14)	0.09
**Coabundance Factor 3[b] (unitless)**	3.6 (-0.87, 8.0)	0.11	-2.0 (-8.7, 4.7)	0.55
**Mother's age (years)**	-0.39 (-0.77, -0.02)	0.04*	-0.46 (-1.0, 0.11)	0.11
**Mother completed high school (yes)**	4.6 (-1.5, 11)	0.13	-0.70 (-9.8, 8.4)	0.88
**Breastfeeding duration (months)**	1.2 (0.30, 2.0)	0.01**	-0.45 (-1.8, 0.86)	0.49
**Child sex (male)**	0.47 (-4.6, 5.5)	0.85	-11 (-18, -2.9)	0.008**
**Child's age (months)**	-1.3 (-5.9, 3.3)	0.57	3.9 (-3.0, 11)	0.26
**Child consumed fish within previous 24 hour (yes)**	5.6 (-0.81,12)	0.09	12 (2.0, 21)	0.02*
**Child's weight for age (z-score)**	2.6 (0.11, 5.0)	0.04*	2.4 (-1.3, 6.1)	0.19
**Child attends preschool (yes)**	3.4 (-2.2, 9.0)	0.23	0.31 (-8.1, 8.7)	0.94
**Birth mode (Cesarean = yes)**	-1.1 (-6.5, 4.4)	0.69	2.4 (-5.8, 11)	0.56
**Child illness in the previous 12 months (yes)[2]**	2.7 (-3.1, 8.5)	0.35	3.7 (-5.0, 12)	0.39

REFERENCES

Klindworth, A., et al. Evaluation of general 16S ribosomal RNA gene PCR primers for classical and next-generation sequencing-based diversity studies. *Nucleic Acids Res.* **41**, 10.1093/nar/gks808 (2013).

World Health Organization (WHO) Expert Consultation. Appropriate body-mass index for Asian populations and its implications for policy and intervention strategies. *Lancet.* **363**, 157-163 (2004).
